# Systematic study on the dependence of the warm-start quantum approximate optimization algorithm on approximate solutions

**DOI:** 10.1038/s41598-023-50406-8

**Published:** 2024-01-12

**Authors:** Ken N. Okada, Hirofumi Nishi, Taichi Kosugi, Yu-ichiro Matsushita

**Affiliations:** 1 Center for Quantum Information and Quantum Biology, Osaka University, Osaka, 560-8531 Japan; 2 Laboratory for Materials and Structures, Institute of Innovative Research, Tokyo Institute of Technology, Tokyo, 152-8550 Japan; 3 Quemix Inc., Tokyo, 103-0027 Japan; 4 Quantum Material and Applications Research Center, National Institutes for Quantum Science and Technology, Tokyo, 152-8552 Japan

**Keywords:** Quantum information, Quantum simulation

## Abstract

Quantum approximate optimization algorithm (QAOA) is a promising hybrid quantum-classical algorithm to solve combinatorial optimization problems in the era of noisy intermediate-scale quantum computers. Recently it has been revealed that warm-start approaches can improve the performance of QAOA, where approximate solutions are obtained by classical algorithms in advance and incorporated into the initial state and/or unitary ansatz. In this work, we study in detail how the accuracy of approximate solutions affects the performance of the warm-start QAOA (WS-QAOA). We numerically find that in typical MAX-CUT problems, WS-QAOA achieves higher fidelity (probability that exact solutions are observed) and approximation ratio than QAOA as the Hamming distance of approximate solutions to the exact ones becomes smaller. We reveal that this could be quantitatively attributed to the initial state of the ansatz. We also solve MAX-CUT problems by WS-QAOA with approximate solutions obtained via QAOA, having higher fidelity and approximation ratio than QAOA especially when the circuit is relatively shallow. We believe that our study may deepen understanding of the performance of WS-QAOA and also provide a guide as to the necessary quality of approximate solutions.

## Introduction

The last decade has seen significant technological progress in manufacturing hardware platform of quantum computers^1^. The current pace of scale-up in quantum devices raises a hope that quantum processors with hundreds or thousands of physical qubits could be available within the next decade. These near-term quantum computers are referred to as noisy intermediate-scale quantum (NISQ) computers^2^ in that they are classically intractable, but still not sufficiently large to implement quantum error correction. As the NISQ era approaches, there have been an increasing number of researches that develop algorithms to efficiently leverage NISQ devices^3–5^. They are designed to solve quantum many-body problems in chemistry and physics as well as classical problems in combinatorial optimization and machine learning. Most of these studies employ hybrid quantum-classical approaches, primarily variational quantum algorithms^6,7^. In these algorithms, variational quantum states are created via parametrized quantum circuits on a quantum computer, whereas the parameters are updated on a classical computer to optimize the objective function calculated with the measurement outcomes. Since variational quantum algorithms generally take a relatively low number of gate operations, they are considered as suitable to gain quantum advantage on NISQ computers.

Quantum approximate optimization algorithm (QAOA)^8^, a representative example of variational quantum algorithms, solves combinatorial optimization problems in a spirit analogous to adiabatic quantum annealing (QA)^9–11^. The variational state (ansatz) of QAOA can be deduced by applying the Trotter decomposition to the time evolution of QA and allowing the time step of each term to vary. Despite some numerical demonstrations of its efficacy in small-size problems^12,13^, it has been a subject of discussions whether QAOA could practically outperform the best classical algorithms^14,15^. Several kinds of variants have been proposed to improve upon the original version of QAOA^16–22^. To name a few, Farhi et al. proposed a variant of the ansatz by allowing different parameters for each rotation gate^16^. Hadfield et al. extended the ansatz by generalizing mixer operations, which could be suitable to optimization problems with constraints^19^.

A promising variant that our work will focus on is the warm-start QAOA (WS-QAOA) proposed by Egger et al.^21^ and Tate et al.^22^. The warm-start approach adjusts the distribution of bit strings in the initial state of the ansatz away from the equal superposition for standard QAOA to increase the amplitude of a classically-obtained approximate solution. Both of Refs.^21^ and^22^ showed enhancement of the solution quality relative to the original version of QAOA particularly for small depth. Egger et al. encode rounded/unrounded semidefinite programming solutions into the initial state as well as the mixer term in the ansatz^21^. They numerically showed improvement in the optimized energy and fidelity compared to standard QAOA in portfolio optimization problems by warm-starting QAOA with classically-obtained continuous solutions. They also solved MAX-CUT problems by WS-QAOA with rounded solutions produced by the Goemans-Williamson algorithm and observed maximum cuts more often than standard QAOA in the recursive procedure developed in Ref.^15^. Tate et al. also encode semidefinite programming relaxations into the initial state, but not the unitary circuit^22^. They observed improvement in the approximation ratio relative to standard QAOA for MAX-CUT problems on weighted and unweighted graphs by using WS-QAOA with relaxed solutions obtained by the Burer-Monteiro algorithm. We also mention that a similar warm-start approach has been independently studied in QA^23^. Meanwhile, it has been reported that WS-QAOA typically shows no improvement if the initial state is strictly a classical string^24^.

In this paper, we examine how the performance of WS-QAOA depends on quality of approximate solutions to make a deep understanding of its efficacy. In Refs.^21,22^, the authors solved problems with WS-QAOA by acquiring approximate solutions by classical algorithms. Meanwhile, it remains unclear how accurate approximate solutions should be for WS-QAOA to outperform QAOA. Here we deduce the ansatz of WS-QAOA starting from QA with a bias field^23^ and carefully study how the performance of WS-QAOA depends on the quality of approximate solutions by numerical simulations on the MAX-CUT problem. We find out that WS-QAOA shows higher fidelity and approximation ratio than QAOA as the Hamming distance of the approximate solutions to the exact ones becomes smaller. We also reveal that the observation could be partially attributed to the initial state of the ansatz. Finally, we solve the MAX-CUT problem with WS-QAOA after obtaining approximate solutions by QAOA and have higher fidelity and approximation ratio than QAOA especially when the circuit depth is small.

The rest of the paper is organized as follows. In Sect. 2, we formulate WS-QAOA in the context of QA. Then, in Sect. 3, we numerically study the performance of WS-QAOA on the MAX-CUT problem for various approximate solutions in terms of the Hamming distance to the exact solutions as well as for different strengths of the bias field. In Sect. 4, we solve the MAX-CUT problem by combining WS-QAOA with QAOA and compare its efficacy to QAOA. Finally, in Sect. 5, we summarize our results.

## Formulation

### MAX-CUT problem

As a prototypical combinatorial optimization problem, we consider the MAX-CUT problem, which is known as NP-hard. It is defined on a graph $G=(V, E)$, where *V* represents a set of vertices, and *E* represents a set of edges between the vertices. We denote the number of vertices in *G* as *n*. The MAX-CUT problem is to find a partition of *V* into two subsets that maximizes the total number of edges between one subset and the other. In a general case that each edge is associated with a real-valued weight $w_{ij}$, one evaluates the weighted sum of those edges. The problem is formulated as maximization of the following objective function

1 $$\begin{aligned} C(\{x_i\})=\sum _{(i,j)\in E}w_{ij}(x_i(1-x_j)+x_j(1-x_i)), \end{aligned}$$

where $x_i$ denotes a binary variable associated with vertex *i* $(x_i=0, 1)$. We note that bit strings $\{x_i\}$ and $\{{\overline{x}}_i\}$ (${\overline{x}}_i\equiv 1-x_i$) give the same value of *C*. In the following, we denote $\{x^{\rm sol}_i\}$ and $\{{\overline{x}}^{\rm sol}_i\}$ as the single pair of solutions.

In the language of physics, the MAX-CUT problem is encoded in finding the ground state of the corresponding Ising Hamiltonian, which is obtained by replacing $x_i$ for $(1-Z_i)/2$ (*Z*: the Pauli *Z* matrix) in the objective function *C* and changing the whole sign. The Hamiltonian reads

2 $$\begin{aligned} H_{\rm C}=\sum _{(i,j)\in E}\frac{w_{ij}}{2}Z_iZ_j, \end{aligned}$$

where the constant term

3 $$\begin{aligned} D=-\sum _{(i,j)\in E}\frac{w_{ij}}{2} \end{aligned}$$

is left off.

### QAOA

QAOA searches for the ground state of the Hamiltonian $H_{\rm C}$ using a QA-inspired ansatz with 2*p* variational parameters for depth *p*^8^. The ansatz is constructed by alternating applications of the driver operation $U_{\rm C}$ and mixer operation $U_{\rm T}$ to the equal-weight superposition state $\left| + \right\rangle ^{\otimes n}$. It is written down with variational parameters $\beta _s$ and $\gamma _s$ $(1\le s\le p)$ as

4 $$\begin{aligned} \left| \Psi _{\rm QAOA} \right\rangle =\prod _{s=1}^pU_{\rm T}(\beta _s)U_{\rm C}(\gamma _s)\left| + \right\rangle ^{\otimes n}. \end{aligned}$$

Here the driver and mixer are defined as $U_{\rm C}(\gamma _s)=e^{-i\gamma _s H_{\rm C}}$ and $U_{\rm T}(\beta _s)=e^{-i\beta _s H_{\rm T}}$, respectively, where $H_{\rm T}=-\sum _iX_i$ (*X*: the Pauli *X* matrix) represents a transverse-field term. One can deduce the ansatz $\left| \Psi _{\rm QAOA} \right\rangle$ via the Trotter decomposition of the QA procedure and parametrization of each time step. In QA, the wave function evolves under the Hamiltonian

5 $$\begin{aligned} H_{\rm QA}(t)=(1-u(t))H_{\rm T}+u(t)H_{\rm C} \end{aligned}$$

with a schedule function *u*(*t*) ($u(0)=0$ and $u(T)=1$).

### WS-QAOA

QA has had considerable success in solving combinatorial optimization problems^9–11^. However, when gap closing occurs during the annealing, it often gets stuck at suboptimal solutions. Reverse QA is an effective variant to circumvent this challenge, which incorporates in the annealing process an approximate solution obtained in advance^25^. In this procedure, the state adiabatically evolves from the approximate solution at the beginning to the exact solution at the end, driven by quantum fluctuations of a transverse field with a mountain-like time profile. The dynamics is described by the Hamiltonian $H_{\rm RQA}(t)=(1-t/T)H_{\rm I}+h(t)H_{\rm T}+(t/T)H_{\rm C}$ $(0\le t\le T)$, where $H_{\rm I}$ yields the approximate solution as the ground state, and *h*(*t*) is a concave function with $h(0)=h(T)=0$. It was shown that the performance of reverse QA is largely dominated by the Hamming distance of the approximate solution from the exact one^25^.

Recently Graß^23^ proposed a similar but simpler QA procedure to make use of an approximate solution, which introduces a longitudinal bias field that favors the approximate solution. The procedure, which we call biased quantum annealing (BQA) hereafter, is governed by the Hamiltonian

6 $$\begin{aligned} H_{\rm BQA}(t)=(1-u(t))(H_{\rm T}+H_{\rm L})+u(t)H_{\rm C}, \end{aligned}$$

where $H_{\rm L}$ represents a site-dependent longitudinal field defined as

7 $$\begin{aligned} H_{\rm L}=-\alpha \sum _i\left( 1-2x_i^0\right) Z_i. \end{aligned}$$

Here $x_i^0$ represents the *i*-th bit in the bit string of an approximate solution $\{x_i^0\}$, and $\alpha$ denotes strength of the bias field. The author numerically showed that BQA achieves higher fidelity than QA in small instances of the exact-cover problem when one prepares approximate solutions that are close enough to the exact solutions in terms of the Hamming distance^23^.

Here we formulate a QAOA version of BQA, which actually corresponds to WS-QAOA^21,22^. One can derive the ansatz via the Trotter decomposition of BQA under the Hamiltonian $H_{\rm BQA}(t)$ and parametrization of each time step in the same manner as one deduces $\left| \Psi _{\rm QAOA} \right\rangle$ from $H_{\rm QA}(t)$. Then the WS-QAOA ansatz is represented as

8 $$\begin{aligned} \left| \Psi _\mathrm{WS-QAOA} \right\rangle =\prod _{s=1}^pU_{\rm T}(\beta _s)U_{\rm L}(\beta _s)U_{\rm C}(\gamma _s)\left| \Psi _0 \right\rangle , \end{aligned}$$

where $U_{\rm L}$ is defined as $U_{\rm L}(\beta _s)=e^{-i\beta _s H_{\rm L}}$. The initial state $\left| \Psi _0 \right\rangle$ is written down as

9 $$\begin{aligned} \left| \Psi _0 \right\rangle =\prod _{i=1}^n\otimes R_Y\left( -\frac{\pi }{2}+\left( 1-2x_i^0\right) \tan ^{-1}\alpha \right) \left| 0 \right\rangle . \end{aligned}$$

$R_Y(\theta )=e^{i(\theta /2)Y}$ (*Y*: the Pauli *Y* matrix) represents a $\theta$-rotation around the *y*-axis. For $\alpha =0$, $\left| \Psi _\mathrm{WS-QAOA} \right\rangle$ corresponds to the QAOA ansatz $\left| \Psi _{\rm QAOA} \right\rangle$. The WS-QAOA ansatz $\left| \Psi _\mathrm{WS-QAOA} \right\rangle$ is almost identical to that in Ref.^21^ except a small difference in representation of the mixer; the latter implements $e^{-i\beta _s (H_{\rm T}+H_{\rm L})}$ with three layers of rotation gates, whereas the former uses a decomposed form $e^{-i\beta _s H_{\rm T}}e^{-i\beta _s H_{\rm L}}$ with two layers.

## Numerical simulations

In this section, we examine how the WS-QAOA performance varies with choice of approximate solutions $\{x_i^0\}$. For that purpose, we numerically study the MAX-CUT problem on weighted 3-regular (w3R) graphs^13,26^. In w3R graphs, each vertex is connected to three others chosen at random, and each edge has weight $w_{ij}$ randomly set from [0, 1). We employ a fast quantum circuit simulator Qulacs^27^.

For optimization of the parameters, we use two methods, random initialization (RI) and an interpolation-based heuristic termed INTERP^13^. In RI, we take the best sample out of 50 randomizations of the initial values. Given the translational symmetry of the ansatz $\left| \Psi _\mathrm{WS-QAOA} \right\rangle$, initial values of $\beta _s$ are set from $[-\frac{\pi }{4}, \frac{\pi }{4})$ for $\alpha =0$, $[-\frac{\pi }{2}, \frac{\pi }{2})$ for $\alpha =1$, and $[-\pi , \pi )$ otherwise, whereas those of $\gamma _s$ are set from $[-2\pi , 2\pi )$. On the other hand, in INTERP, the parameters are optimized incrementally from depth 1 to depth *p*. Here initial values of the parameters at depth *p*, $\beta _s[p]$ and $\gamma _s[p]$ $(1\le s\le p)$, are uniquely determined via an interpolation of the optimized values at depth $p-1$ as $\beta _s[p]=\frac{s-1}{p-1}\beta _{s-1}[p-1]+\left( 1-\frac{s-1}{p-1}\right) \beta _s[p-1]$ ($\beta _0[p-1]=\beta _p[p-1]=0$). It has been revealed that INTERP works more efficiently than RI for QAOA on w3R graphs^13^. In this work, based on our benchmark calculations, we choose a better method, depending on $\alpha$, *p*. For QAOA ($\alpha =0$), we use INTERP. For WS-QAOA, with $\alpha =0.4$, we use INTERP, whereas, with $\alpha =1$, we use RI at $p\le 3$ and INTERP at $p=4$. We note that, regardless of $\alpha (\ne 0)$, we use RI when $\{x_i^0\}$ corresponds to the exact solution. In large instances of practical interest where exact solutions are unknown, one would choose either of the methods only according to $\alpha$ and *p*. In both methods, parameters are updated via a gradient descent until the gradient becomes lower than a certain threshold value.

**Figure 1:**
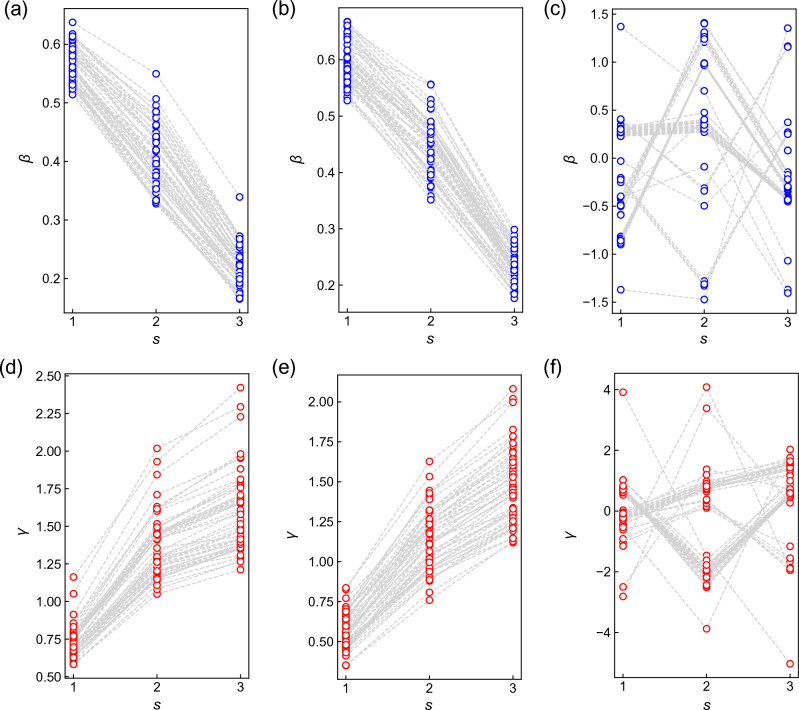
Optimized $\beta _s$ and $\gamma _s$ of (**a**,**d**) QAOA and WS-QAOA with (**b**,**e**) $\alpha =0.4$ and (**c**,**f**) $\alpha =1$ for $d=1$ on 50 instances of w3R graph ($n=10$). The ansatz depth is $p=3$. The parameters are optimized by INTERP in (**a**,**b**,**d**,**e**) and RI in (**c**,**f**).

In WS-QAOA, we set approximate solutions $\{x_i^0\}$ by flipping *d* bits randomly selected from *n* bits in the solution $\{x_i^{\rm sol}\}$. In other words, *d* represents the Hamming distance of $\{x_i^0\}$ to $\{x^{\rm sol}_i\}$. Figure 1 show the optimized parameters of QAOA $(\alpha =0)$, and WS-QAOA with $\alpha =0.4, 1$ for $d=1$ on 50 graph instances of $n=10$. Fig. 1a,d show that in QAOA, $\beta _s$ ($\gamma _s$) decreases (increases) with *s*, which resembles the process of QA^13^. We observe a similar trend in WS-QAOA with $\alpha =0.4$ in Fig. 1b, e. Meanwhile, when $\alpha =1$, the parameters are not monotonic against *s*, which may reflect that the property of QA declines as $\alpha$ becomes larger. Moreover, the optimized parameters do not seem transferable between instances for $\alpha =1$ (Fig. 1c,f) in comparison to those for QAOA (Fig. 1a,d) and $\alpha =0.4$ (Fig. 1b,e). We think that further studies would be needed on parameter transferability^28,29^ for WS-QAOA.

**Figure 2:**
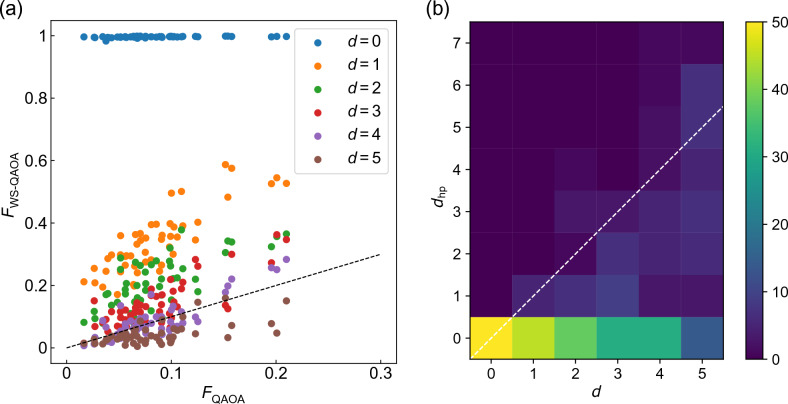
Performance of WS-QAOA with $\alpha =0.4$ at $p=3$ on 50 instances of w3R graph ($n=14$). *d* represents the Hamming distance between the approximate solution $\{x^0_i\}$ and exact one $\{x^{\rm sol}_i\}$. (**a**) Fidelity of WS-QAOA ($F_\mathrm{WS-QAOA}$) versus that of QAOA ($F_{\rm QAOA}$). The dotted line corresponds to $F_\mathrm{WS-QAOA}=F_{\rm QAOA}$. (**b**) Histogram over *d* and $d_{\rm hp}$. $d_{\rm hp}$ is the Hamming distance of $\{x^{\rm hp}_i\}$ to $\{x^{\rm sol}_i\}$. The dotted line corresponds to $d_{\rm hp}=d$.

We compare the performance of WS-QAOA to that of QAOA. As a performance indicator, we use the fidelity of the optimized ansatz $\left| \Psi _\mathrm{WS-QAOA} \right\rangle$. We define the fidelity of a wave function $\left| \Phi \right\rangle$ as

10 $$\begin{aligned} F=|\left\langle \{x^{\rm sol}_i\}|\Phi \right\rangle |^2+|\left\langle \{{\overline{x}}^{\rm sol}_i\}|\Phi \right\rangle |^2. \end{aligned}$$

Later, we also discuss our results in terms of approximation ratio, defined as $r=-(\left\langle \Phi |H_{\rm C}|\Phi \right\rangle +D)/C(\{x^{\rm sol}_i\})$. In Fig. 2a, *F* of WS-QAOA with $\alpha =0.4$ at $p=3$ is plotted against that of QAOA on 50 graph instances of $n=14$. In the following, we focus on $\alpha =0.4, 1$. We refer to Sect. I of SM for a closer look at $\alpha$ dependence. Figure 2a indicates that the relative performance of WS-QAOA against QAOA is dominated by the Hamming distance *d*. Importantly, $F_\mathrm{WS-QAOA}$ becomes higher as *d* decreases. We find that WS-QAOA outperforms QAOA in all cases for $d\le 2$ and in most cases for $d=3$ (Fig. 2a). We note that $F_\mathrm{WS-QAOA}$ is always almost unity for $d=0$. The enhanced fidelity with the decrease in *d* has also been observed in BQA^23^.

Success of WS-QAOA with small *d* is also manifested in the bit string with highest probability, $\{x^{\rm hp}_i\}$, in the optimized ansatz. Figure 2b shows the histogram over *d* and the Hamming distance of $\{x^{\rm hp}_i\}$ to $\{x^{\rm sol}_i\}$, $d_{\rm hp}$, on 50 instances of $n=14$. One can see that the closer the approximate solution is to the exact solution, the more likely $\{x^{\rm hp}_i\}$ is to correspond with the exact solution ($d_{\rm hp}=0$) (Fig. 2b). It is notable that for $d=4$, the optimized ansatz still yields the solution as the highest-probability string in about a half of the instances.

**Figure 3:**
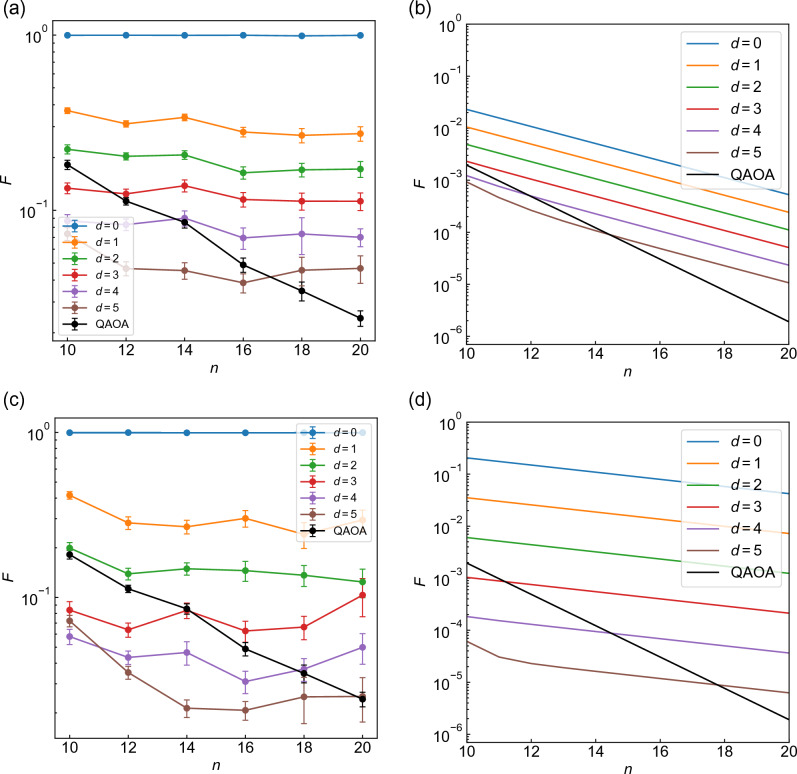
(**a**,**c**) Graph size dependence of the average fidelity obtained by WS-QAOA with different *d* compared to QAOA ($\alpha =0$) for $p=3$. (**a**) corresponds to $\alpha =0.4$ and (**c**) to $\alpha =1$. The fidelity is averaged over 50 instances for $n\le 14$, 20 for $n=16$, 15 for $n=18$, and 10 for $n=20$. The error bar represents standard error of the mean. (**b**,**d**) Calculated fidelity for the initial state of the ansatz, Eq. (11). (**b**) corresponds to $\alpha =0.4$, (**d**) to $\alpha =1$ and black lines to QAOA ($\alpha =0$).

We proceed to study the graph size dependence. In Fig. 3a,c, the averaged fidelity of WS-QAOA at $p=3$ is shown against the number of vertices *n*, together with that of QAOA ($\alpha =0$). Figure 3a,c correspond to $\alpha =0.4$ and $\alpha =1$, respectively. We present the entire data for $p=1-4$ in Sect. II of SM. In both WS-QAOA and QAOA, *F* shows a nearly exponential decay with *n*, but importantly it decreases less steeply in WS-QAOA than in QAOA. As a result, with larger *n*, WS-QAOA outperforms QAOA with even larger *d*. We also compare $\alpha =0.4$ and $\alpha =1$. Figure 3a,c indicate that as *d* increases incrementally, fidelity decreases roughly by a constant multiplicative factor (aside from $d=0\rightarrow 1$) and that the factor is smaller for $\alpha =0.4$ than for $\alpha =1$. These features seem to stem from the initial state at least in part. In Fig. 3b,d, we present the fidelity of $\left| \Psi _0 \right\rangle$ for $\alpha =0.4$ and $\alpha =1$, which is derived from Eqs. (10) and (9) with $\left| \Phi \right\rangle =\left| \Psi _0 \right\rangle$ as

11 $$\begin{aligned} \begin{aligned} F_0&=\cos ^{2d}\left( \frac{\pi }{4}+\frac{\tan ^{-1}\alpha }{2}\right) \cos ^{2(n-d)}\left( \frac{\pi }{4}-\frac{\tan ^{-1}\alpha }{2}\right) \\&+\cos ^{2d}\left( \frac{\pi }{4}-\frac{\tan ^{-1}\alpha }{2}\right) \cos ^{2(n-d)}\left( \frac{\pi }{4}+\frac{\tan ^{-1}\alpha }{2}\right) . \end{aligned} \end{aligned}$$

In Fig. 3b,d, one can observe similar behaviors to Fig. 3a,c, although the magnitude of the fidelity is significantly improved by the optimized circuit.

**Figure 4:**
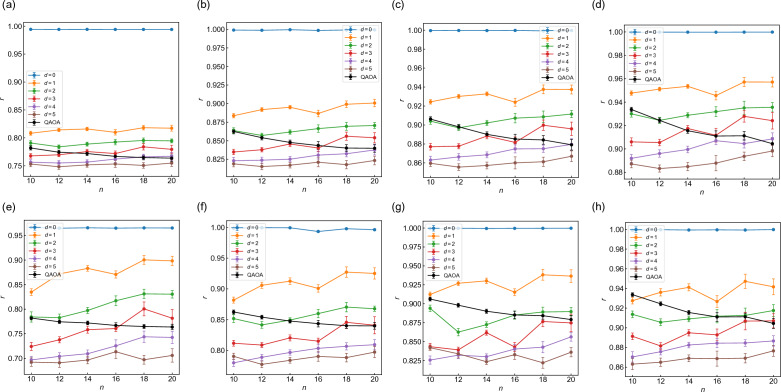
Graph size dependence of the approximation ratios of WS-QAOA along with QAOA (**a**–**d**) $\alpha =0.4$ and (**e**-**h**) $\alpha =1$. (**a**) and (**e**) correspond to $p=1$, (**b**) and (**f**) to $p=2$, (**c**) and (**g**) to $p=3$, and (**d**) and (**h**) to $p=4$. The approximation ratio is averaged over 50 graph instances for $n\le 14$, 20 for $n=16$, 15 for $n=18$, and 10 for $n=20$. The error bar stands for standard error of the mean.

We also present the *n* dependence of approximation ratio *r* with $p=1-4$ in Fig. 4. Figure 4a–d correspond to $\alpha =0.4$, and Fig. 4e–h to $\alpha =1$. As in the case of fidelity *F* (Fig. 3), WS-QAOA has higher *r* than QAOA when the Hamming distance *d* is sufficiently small. *r* of WS-QAOA also decreases as *d* increases. Notably, *r* of WS-QAOA tends to increase with *n* as opposed to *F*.

**Figure 5:**
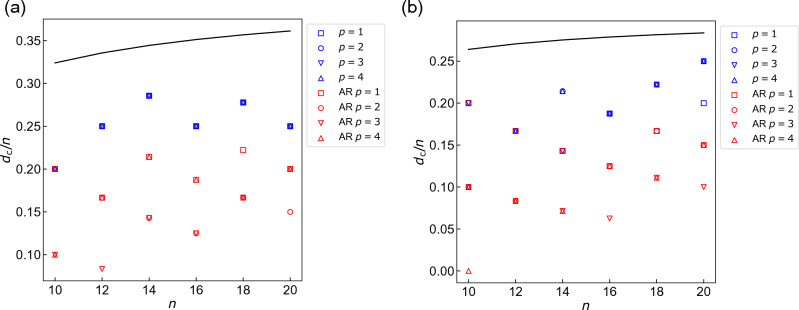
Critical relative Hamming distance $d_{\rm c}/n$ plotted against *n* for (**a**) $\alpha =0.4$ and (**b**) $\alpha =1$. The blue points are derived from the fidelity in Fig. 3a,c, and S2, and red ones are from the approximation ratio in Fig. 4. When approximate solutions are less-than-$d_{\rm c}$ away from exact solutions, WS-QAOA outperforms QAOA on average. The black line denotes the theoretical value of $d_{\rm c}/n$ estimated for the fidelity of the initial state $\left| \Psi _0 \right\rangle$ (see Eq. (12) in the text).

The calculations above indicate how close approximate solutions should be to the exact solutions for WS-QAOA to outperform QAOA. From the graph size dependence of the fidelity (Fig. 3a,c, and S2) as well as the approximation ratio (Fig. 4), we estimate the critical Hamming distance of $\{x^0_i\}$, $d_{\rm c}$, which determines whether WS-QAOA outperforms QAOA or not. For example, we estimate $d_{\rm c}=3$ for $n=12, \alpha =0.4$ from the fidelity data in Fig. 3a. Figures 5a and 5b show $d_{\rm c}$ scaled by the graph size *n* for $\alpha =0.4$ and $\alpha =1$, respectively. The blue (red) points represent $d_{\rm c}/n$ for fidelity (approximation ratio). For fidelity, $d_{\rm c}/n$ ranges within [0.2, 0.3) for $\alpha =0.4$, whereas it hovers from 0.1 to 0.25 for $\alpha =1$. For approximation ratio, $d_{\rm c}/n$ is smaller, mostly within [0.1, 0.25) for $\alpha =0.4$ and [0.05, 0.2] for $\alpha =1$. The important implication with respect to scalability is that the Hamming distance *d* could be allowed to scale linearly with *n* when *d*/*n* is below those threshold values.

We also theoretically derive $d_{\rm c}/n$ for the fidelity of the initial state $\left| \Psi _0 \right\rangle$ starting from $F_0(\alpha )=F_0(\alpha =0)$ (see Sect. III of SM), which reads

12 $$\begin{aligned} \begin{aligned} \frac{d_{\rm c}}{n}&=\frac{\log \left( \cos \frac{\pi }{4}/\cos \left( \frac{\pi }{4}-\frac{\tan ^{-1}\alpha }{2}\right) \right) }{\log \tan \left( \frac{\pi }{4}-\frac{\tan ^{-1}\alpha }{2}\right) }\\&+\frac{1}{2n}\frac{\log \left( 1+\sqrt{1-\sin ^{2n}\left( \frac{\pi }{2}-\tan ^{-1}\alpha \right) }\right) }{\log \tan \left( \frac{\pi }{4}-\frac{\tan ^{-1}\alpha }{2}\right) }. \end{aligned} \end{aligned}$$

We draw the curve of Eq. (12) in Figs. 5. One can see that in both $\alpha$, $d_{\rm c}/n$ estimated from the actual fidelity is smaller than the theoretical curve for the initial state. This indicates that QAOA gains more fidelity by the optimized unitary circuit than WS-QAOA.

**Figure 6:**
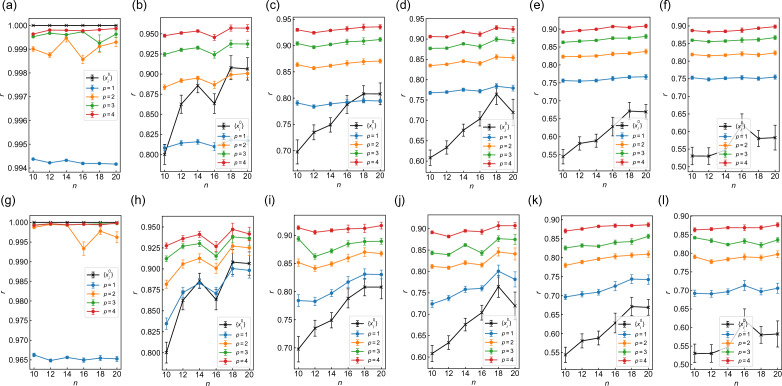
Graph size dependence of the approximation ratios of WS-QAOA as well as approximate solutions employed $\{x_i^0\}$ for (**a**–**f**) $\alpha =0.4$ and (**g**–**l**) $\alpha =1$. (**a**) and (**g**) correspond to $d=0$, (**b**) and (**h**) to $d=1$, (**c**) and (**i**) to $d=2$, (**d**) and (**j**) to $d=3$, (**e**) and (**k**) to $d=4$, (**f**) and (**l**) to $d=5$. The approximation ratio is averaged over 50 graph instances for $n\le 14$, 20 for $n=16$, 15 for $n=18$, and 10 for $n=20$. The error bar stands for standard error of the mean.

We also study how much or whether WS-QAOA improves approximation ratio as compared to approximate solutions employed in WS-QAOA. Figure 6 display the graph size dependence of approximation ratio *r* for WS-QAOA as well as approximate solutions employed $\{x_i^0\}$ with $d=0-5$. Fig. 6a–f correspond to $\alpha =0.4$ and Fig. 6g–l to $\alpha =1$. In most cases, WS-QAOA yields higher approximation ratio on average than approximate solutions for $d\ge 2$ with $p\ge 1$ (Figs. 6c–f, 6i–l) and $d=1$ with $p\ge 2$ (Fig. 6b, 6h). This makes it clear that WS-QAOA helps to improve upon the approximate solutions obtained in advance.

**Figure 7:**
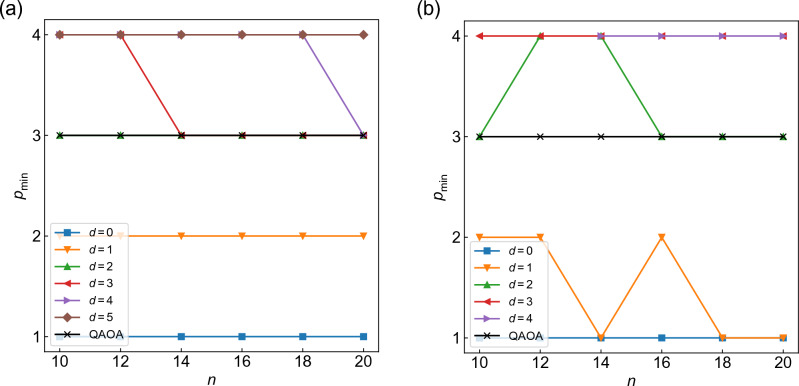
Minimal *p* that reaches the target approximation ratio $r_{\rm t}=0.878$ plotted as a function of *n* for WS-QAOA with (**a**) $\alpha =0.4$ and (**b**) $\alpha =1$.

Finally, to see the scalability of the methods, we examine the minimal depth *p* that reaches a target approximation ratio, $p_{\min }$. Here we set the target approximation ratio as $r_{\rm t} = 0.878$, the value guaranteed by the classical Goemans-Williamson algorithm for MAX-CUT problems^30^. In Figs. 7a and 7b, we plot $p_{\min }$ as a function of *n* for WS-QAOA with $\alpha =0.4$ and $\alpha =1$, respectively, along with that for QAOA. Figure 7 show that *p* can be smaller for WS-QAOA with $d\le 1$ than for QAOA to reach the target approximation ratio. Meanwhile, since our data are limited to $n\le 20$ and $p\le 4$ due to finite computational resources, it seems difficult to discuss the size dependence for the vanilla and warm-start approaches from Figs. 7. We expect that one could reveal the scalability by increasing *n* and *p* significantly.

## WS-QAOA combined with QAOA

**Figure 8:**
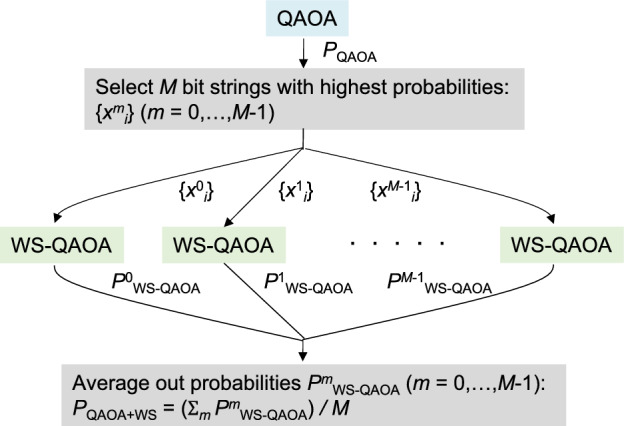
Flow diagram of QAOA+WS.

In the previous section, we studied the dependence of the WS-QAOA performance on approximate solutions and revealed that their Hamming distance to the exact solutions plays a crucial role. In this section, we solve the MAX-CUT problem with WS-QAOA while finding approximate solutions by QAOA. This resembles the approach in the previous study of BQA, where approximate solutions are obtained by QA beforehand^23^.

In Fig. 8, we depict a flow diagram of our procedure. We call this procedure QAOA+WS hereafter. First, we solve the problem using QAOA and pick up *M* bit strings with highest probabilities, $\{x^m_i\}$ ($m=0,...,M-1$), based on the distribution $P_{\rm QAOA}$ from $\left| \Psi _{\rm QAOA} \right\rangle$. Then we conduct WS-QAOA with $\{x^m_i\}$ as an approximate solution and obtain the distribution $P^m_\mathrm{WS-QAOA}$ from $\left| \Psi _\mathrm{WS-QAOA} \right\rangle$. At the end, we obtain the final distribution $P_\mathrm{QAOA+WS}$ by averaging out *M* distributions $P^m_\mathrm{WS-QAOA}$.

**Figure 9:**
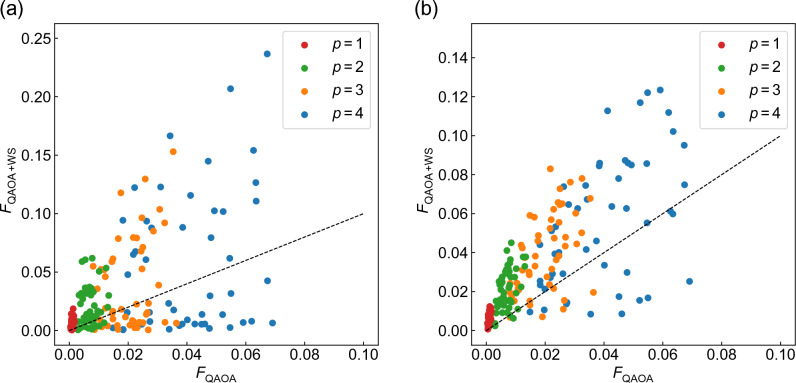
Fidelity of QAOA+WS versus that of QAOA for 50 instances of $n=20$ with (**a**) $M=3$ and (**b**) $M=8$. For WS-QAOA in QAOA+WS, we set $\alpha =0.4$.

We compare the fidelity of QAOA+WS to that of QAOA. The fidelity of QAOA+WS is calculated as the average over the fidelities of *M* runs of WS-QAOA. Figures 9 present fidelities of QAOA+WS with $\alpha =0.4$ against those of QAOA over 50 graph instances of $n=20$. In Fig. 9a,b, we set $M=3$ and $M=8$ for QAOA+WS, respectively. We note that if the exact solutions are included in *M* approximate solutions, we drop them off, considering that $d=0$ almost always yields the perfect fidelity (Figs. 2a, 3a). For $M=3$, QAOA+WS shows a sizable variance of the fidelity especially as *p* increases. It shows a better performance for most instances than QAOA at $p=1$, but not necessarily at $p\ge 2$. Meanwhile, for $M=8$, QAOA+WS shows a smaller variance and outperforms QAOA at $p\le 3$ for most instances. Smaller variance with larger *M* seems to be natural, because the approximate solutions are more likely to have a wide range of the Hamming distance to the exact solutions as *M* increases.

**Figure 10:**
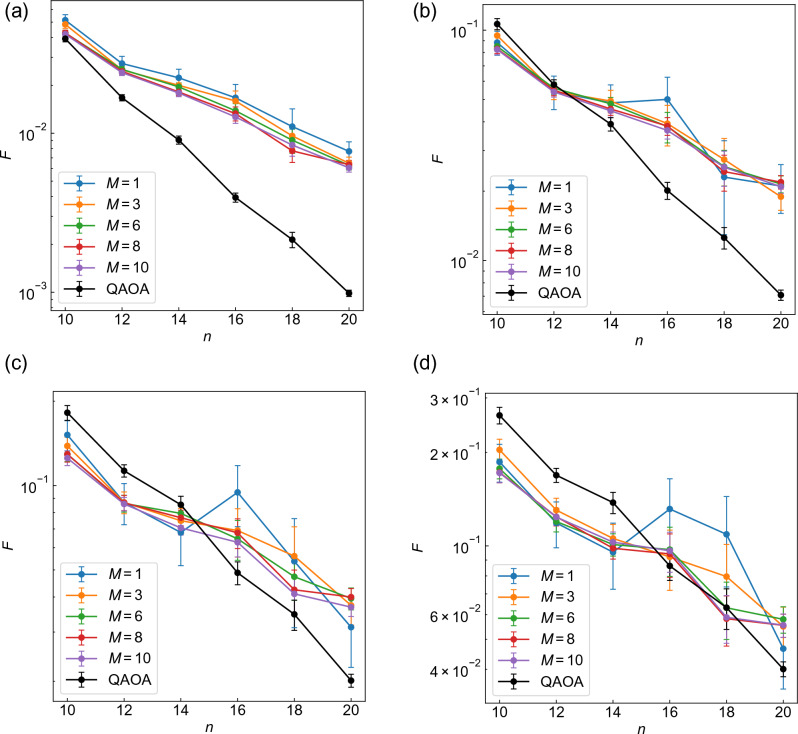
Graph size dependence of the fidelities of QAOA+WS along with QAOA at (**a**–**d**) $p=1-4$. The fidelity is averaged over 50 graph instances for $n=10, 12, 14, 20$, 20 for $n=16$, and 15 for $n=18$. For WS-QAOA in QAOA+WS, we set $\alpha =0.4$. The error bar represents standard error of the mean.

We also study the graph size dependence. Figure 10 present the fidelity of QAOA+WS with $\alpha =0.4$ along with that of QAOA plotted against the number of vertices. In Fig. 10a–d, we set $p=1-4$, respectively. The fidelity is averaged over 50 graph instances for $n=10, 12, 14, 20$, 20 for $n=16$, and 15 for $n=18$. Importantly, the fidelity decays more slowly with *n* in QAOA+WS than in QAOA for $p=1-4$ (Fig. 10). As a result, QAOA+WS on average outperforms QAOA for all *M* as *n* increases; for $n\ge 10$ at $p=1$, $n\ge 14$ at $p=2$, $n\ge 16$ at $p=3$, and $n=20$ at $p=4$. It should be also mentioned that QAOA+WS becomes more beneficial for smaller *p*, because the difference in the decay with *n* seems to decrease as *p* increases.

**Figure 11:**
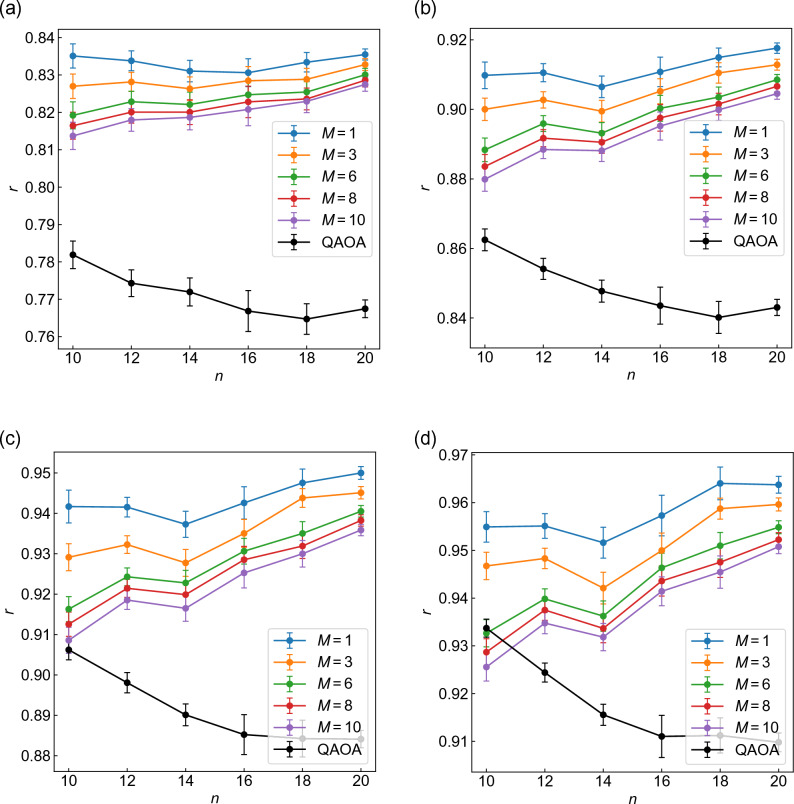
Graph size dependence of the approximation ratios of QAOA+WS along with QAOA at (**a**–**d**) $p=1-4$. The approximation ratio *r* is averaged over 50 graph instances for $n=10, 12, 14, 20$, 20 for $n=16$, and 15 for $n=18$. For WS-QAOA in QAOA+WS, we set $\alpha =0.4$. The error bar represents standard error of the mean.

We also evaluate the performance of QAOA+WS by approximation ratio. As in the fidelity, the approximation ratio *r* for QAOA+WS is estimated by averaging out *r* for *M* runs of WS-QAOA. Figure 11a–d show the graph size dependence of *r* for $p=1-4$, respectively. QAOA+WS achieves higher *r* than QAOA except for $n=10, p=4$. As in the fidelity (Fig. 10), the difference in *r* increases with *n* and decreases as *p* increases. It should be noted that *r* of QAOA+WS has a tendency to increase with *n* as opposed to *F* (Fig. 10).

**Figure 12:**
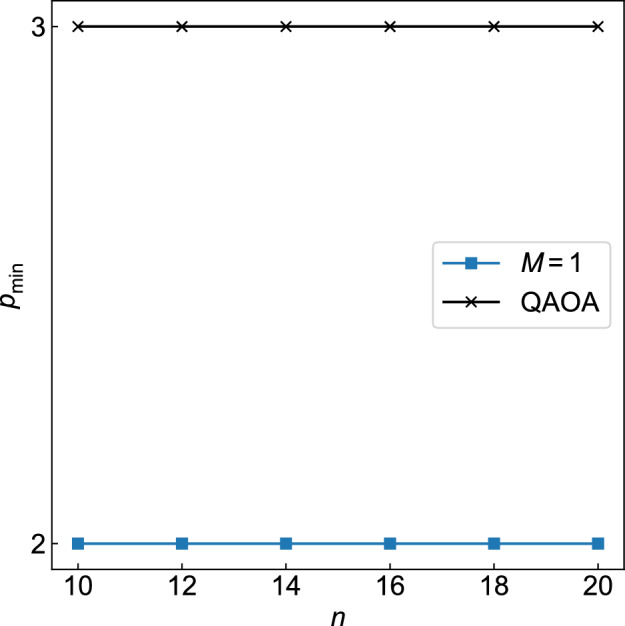
Minimal depth *p* to reach the target approximation ratio $r_{\rm t}=0.878$ plotted as a function of *n* for QAOA+WS with $M=1$ and QAOA.

We present the minimal depth for the target approximation quality for QAOA+WS. Figure 12 shows $p_{\rm min}$ for QAOA+WS with $M=1$ as well as QAOA as a function of *n* (see Sect. 3 for the definition of $p_{\rm min}$). One can see that the minimal *p* is smaller for QAOA+WS than for QAOA (Fig. 12). We note that, as in Fig. 7, it is hard to discuss the scalability of the results due to the limited range of *n* and *p*.

As indicated in Fig. 12, QAOA+WS needs lower circuit depth to reach certain solution qualities than QAOA. Meanwhile, we should remember that QAOA+WS needs more variational circuits than QAOA, where QAOA+WS involves $(M+1)$ circuits ($2p(M+1)$ variational parameters) in comparison to a single circuit (2*p* variational parameters) for QAOA (Fig. 8). In small instances, one may rather use QAOA than QAOA+WS because $p_{\rm min}$ might be small enough for QAOA to be reliably executed on current NISQ devices. However, $p_{\rm min}$ would be much larger for large problems of practical interest. Then one can expect that it would be more important to reduce the circuit depth than the number of variational circuits, considering that circuit fidelity exponentially decreases with depth on NISQ devices due to hardware noise. In such cases, QAOA+WS could be more advantageous than QAOA.

It should be mentioned that approximate solutions fed to WS-QAOA can be obtained by classical solvers instead of QAOA as in Refs.^21,22^. When classical methods give better solution qualities than QAOA, there is not much reason for employing QAOA to obtain approximate solutions. Even when classical methods give solution qualities comparable to QAOA, using classical methods for approximate solutions might be still preferable because it saves quantum resources. In light of these considerations, QAOA+WS would be beneficial in cases that one intends to improve upon QAOA when QAOA already provides better solution qualities than classical methods in the future.

## Conclusion

In this work, we systematically studied how the performance of WS-QAOA depends on the quality of approximate solutions by numerical simulations on the MAX-CUT problem on w3R graphs. We found that WS-QAOA yields higher fidelities and approximation ratios than QAOA when one uses approximate solutions that are close enough to the exact solutions in terms of the Hamming distance. More specifically, WS-QAOA with $\alpha =0.4$ ($\alpha =1$) produces higher fidelities on average than QAOA if the relative Hamming distance of approximate solutions to the exact ones, $d_{\rm c}/n$, is below 0.2–0.3 (0.1–0.25). We also obtained theoretical curves that explain those properties. Furthermore, we showed that QAOA could serve as a capable way to find approximate solutions for WS-QAOA. We found out that WS-QAOA combined with QAOA shows higher fidelity and approximation ratio than QAOA specifically when the depth is limited to a small number.

We believe that our findings could allow one to make a clear understanding of the efficacy of WS-QAOA. They might also be helpful to determining the criteria of approximate solutions for WS-QAOA. Lastly, we mention several future studies of interest. In Ref.^31^, the authors predicted the circuit depth of standard QAOA to reach a certain approximation ratio for various graph symmetries by machine learning technique. It would be interesting to extend their results to WS-QAOA and investigate the necessary conditions for the Hamming distance of the approximation solution as well as the circuit depth. In the context of industrial applications, it would be also intriguing to see how our results would look like for more practical optimization problems.

### Supplementary Information

Supplementary Information.

## Data Availability

The data generated in this study are available from the corresponding author upon reasonable request.
