# Supplementary material for: Systematic study on the dependence of the warm-start quantum approximate optimization algorithm on approximate solutions
Source: Sci Rep. 2024 Jan 12;14:1167. doi: 10.1038/s41598-023-50406-8 (PMC10786944; doi:10.1038/s41598-023-50406-8)
Supplement: Supplementary file 1 — Supplementary Information. [file 41598_2023_50406_MOESM1_ESM.pdf]

Supplementary Material: Systematic study on the dependence of the warm-start quantum approximate optimization algorithm on approximate solutions

Ken N. Okada,[1] Hirofumi Nishi,[2,3] Taichi Kosugi,[2,3] and Yu-ichiro Matsushita[2,3,4]

[1]*Center for Quantum Information and Quantum Biology, Osaka University, Osaka 560-8531, Japan*
[2]*Laboratory for Materials and Structures, Institute of Innovative Research, Tokyo Institute of Technology, Tokyo, 152-8550, Japan*
[3]*Quemix Inc., Tokyo, 103-0027, Japan*
[4]*Quantum Material and Applications Research Center, National Institutes for Quantum Science and Technology, Tokyo, 152-8552, Japan*

I. α DEPENDENCE OF THE FIDELITY OF WS-QAOA

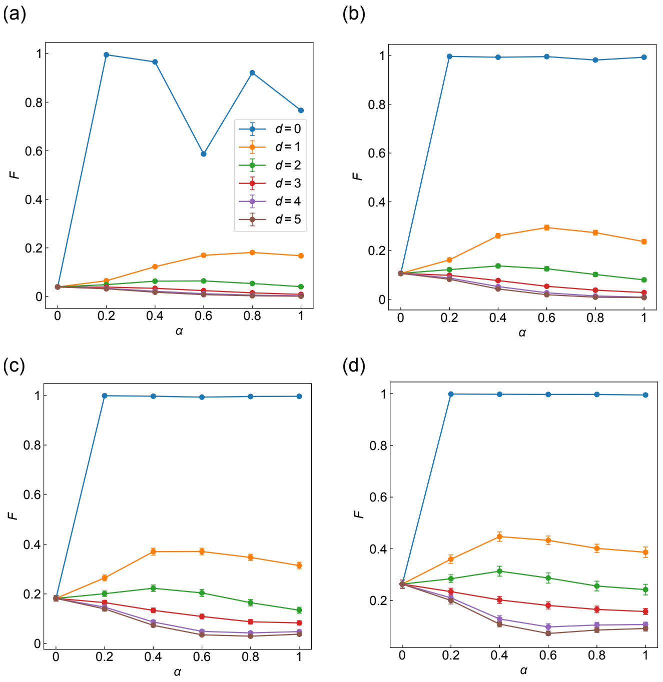

FIG. S1. α dependence of the fidelities for WS-QAOA of $n = 10$ with (a) $p = 1$, (b) $p = 2$, (c) $p = 3$, and (d) $p = 4$. The fidelity is averaged over 50 graph instances. The error bar represents standard error of the mean. For optimization, we use RI for $d = 0$ ($\alpha \neq 0$) and INTERP for the other cases.

We systematically study α dependence of WS-QAOA. Figures S1(a–d) display the averaged fidelity over 50 graph instances of $n = 10$ as a function of α for $p = 1$–4. Here we optimize the parameters using RI for WS-QAOA with $d = 0$ and INTERP otherwise. We note that $\alpha = 0$ corresponds to QAOA. For WS-QAOA with $d = 0$, the fidelity F almost equals 1, aside from $p = 1$. For $d = 1, 2$, F has a peak around $\alpha = 0.4$–0.8, whereas, for $d \geq 3$, F monotonically decreases with α. Therefore the optimal α varies with d.

II. COMPARISON OF THE FIDELITIES OF WS-QAOA WITH DIFFERENT p

We compare the fidelities of WS-QAOA with different values of p. Figures S2(a–d) show the n dependence of the fidelity with $p = 1$–4 for $\alpha = 0.4$. Figures S2(e-h) correspond to $\alpha = 1$. Note that Figs. S2(c) and S2(g) are identical to Figs. 3(a) and 3(c) in the main text. As expected, Figures S2 show that F increases with p for both $\alpha = 0.4$ and $\alpha = 1$. One can see that the tendencies mentioned in Sec. III in the main text are shared among all p. In other words, the fidelity of WS-QAOA decays more slowly with n than that of QAOA and decreases by a multiplicative factor with d, which seems to be larger for $\alpha = 0.4$ than $\alpha = 1$.

III. DERIVATION OF d_c/n FOR THE INITIAL STATE OF THE WS-QAOA ANSATZ

Here we show derivation of Eq. (12). From Eq. (11), $F_0(\alpha) = F_0(\alpha = 0)$ yields

$$\cos^{2d}\left(\frac{\pi}{4}+\frac{\theta}{2}\right)\cos^{2(n-d)}\left(\frac{\pi}{4}-\frac{\theta}{2}\right)+\cos^{2d}\left(\frac{\pi}{4}-\frac{\theta}{2}\right)\cos^{2(n-d)}\left(\frac{\pi}{4}+\frac{\theta}{2}\right)=2\cos^{2n}\left(\frac{\pi}{4}\right), \tag{S1}$$

where we set $\theta = \tan^{-1}\alpha$. With δ defined as $\delta = \tan\left(\frac{\pi}{4}-\frac{\theta}{2}\right)$, Eq. (S1) is represented as

$$\cos^{2n}\left(\frac{\pi}{4}-\frac{\theta}{2}\right)\delta^{2d}+\frac{\cos^{2n}\left(\frac{\pi}{4}+\frac{\theta}{2}\right)}{\delta^{2d}}=2\cos^{2n}\left(\frac{\pi}{4}\right). \tag{S2}$$

We solve Eq. (S2) with respect to δ^{2d} and obtain

$$\delta^{2d}=\frac{\cos^{2n}\left(\frac{\pi}{4}\right)}{\cos^{2n}\left(\frac{\pi}{4}-\frac{\theta}{2}\right)}\left(1\pm\sqrt{1-\sin^{2n}\left(\frac{\pi}{2}-\theta\right)}\right). \tag{S3}$$

Logarithm of Eq. (S3) gives

$$2d\log\delta = 2n\log\frac{\cos\left(\frac{\pi}{4}\right)}{\cos\left(\frac{\pi}{4}-\frac{\theta}{2}\right)}+\log\left(1\pm\sqrt{1-\sin^{2n}\left(\frac{\pi}{2}-\theta\right)}\right). \tag{S4}$$

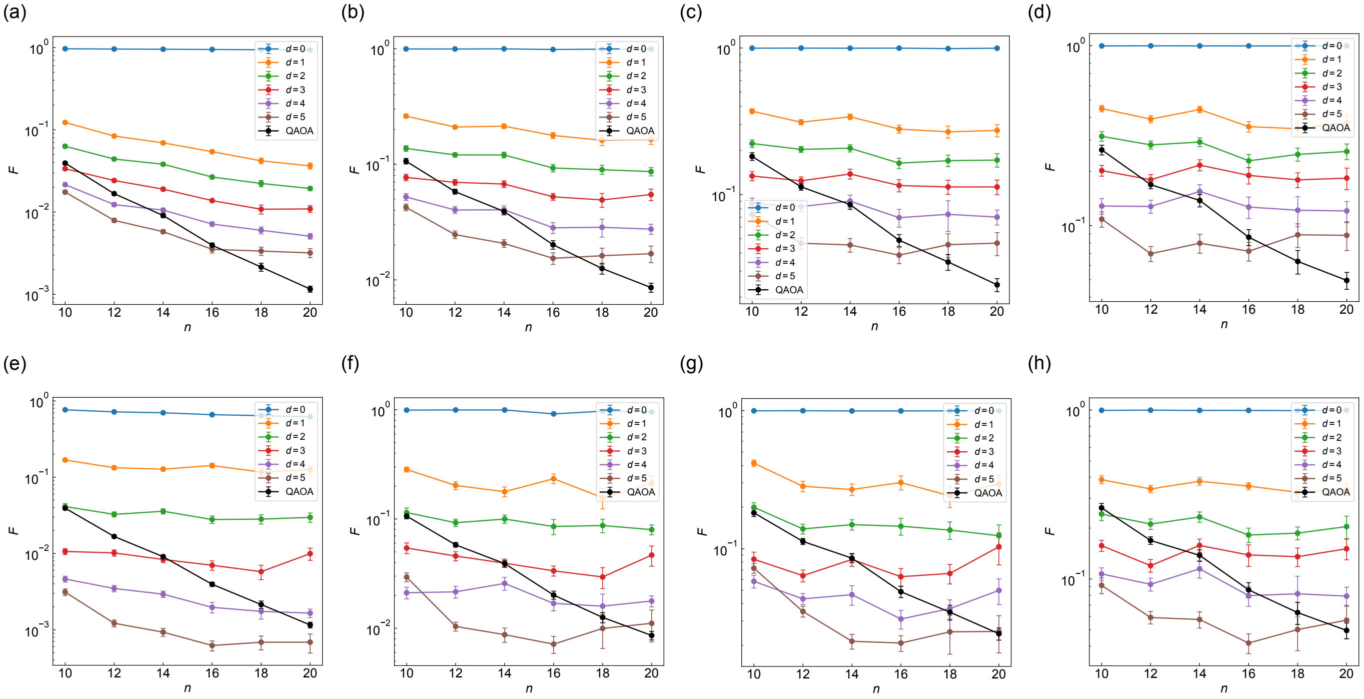

FIG. S2. Graph size dependence of the fidelities of WS-QAOA along with QAOA (a–d) $\alpha = 0.4$ and (e–h) $\alpha = 1$. (a) and (e) correspond to $p = 1$, (b) and (f) to $p = 2$, (c) and (g) to $p = 3$, and (d) and (h) to $p = 4$. The fidelity is averaged over 50 graph instances for $n \leq 14$, 20 for $n = 16$, 15 for $n = 18$, and 10 for $n = 20$. The error bar stands for standard error of the mean.

Thus d/n is derived as

$$\frac{d}{n} = \frac{\log\left(\cos\frac{\pi}{4}/\cos\left(\frac{\pi}{4}-\frac{\theta}{2}\right)\right)}{\log\delta} + \frac{1}{2n}\frac{\log\left(1 \pm \sqrt{1-\sin^{2n}\left(\frac{\pi}{2}-\theta\right)}\right)}{\log\delta}. \tag{S5}$$

As we denote the lefthand side of Eq. (S5) as $d_{\mathrm{c}}^{\pm}/n$ corresponding to $\pm$ in the righthand side, $d_{\mathrm{c}}^{+}/n + d_{\mathrm{c}}^{-}/n = 1$ holds. Considering $0 \leq d \leq n/2$ without loss of generality, $F_0(\alpha) \geq F_0(\alpha = 0)$ holds for $d \leq d_{\mathrm{c}}^{+}$. Hence we represent d_{c}^{+}/n in Eq. (12).
